# Ribonuclease Activity of Dis3 Is Required for Mitotic Progression and Provides a Possible Link between Heterochromatin and Kinetochore Function

**DOI:** 10.1371/journal.pone.0000317

**Published:** 2007-03-21

**Authors:** Hiroaki Murakami, Derek B. Goto, Takashi Toda, Ee Sin Chen, Shiv I. Grewal, Robert A. Martienssen, Mitsuhiro Yanagida

**Affiliations:** 1 CREST Research Program, Japan Science and Technology Corporation, Department of Gene Mechanisms, Graduate School of Biostudies, Kyoto University, Yoshida-Honmachi, Sakyo-ku, Kyoto, Japan; 2 Cold Spring Harbor Laboratory, Cold Spring Harbor, New York, United States of America; 3 Laboratory of Cell Regulation, Cancer Research UK, London Research Institute, Lincoln's Inn Fields Laboratories, London, United Kingdom; 4 Laboratory of Molecular Cell Biology, National Institutes of Health, Bethesda, Maryland, United States of America; Duke University, United States of America

## Abstract

**Background:**

Cellular RNA metabolism has a broad range of functional aspects in cell growth and division, but its role in chromosome segregation during mitosis is only poorly understood. The Dis3 ribonuclease is a key component of the RNA-processing exosome complex. Previous isolation of the *dis3-54* cold-sensitive mutant of fission yeast *Schizosaccharomyces pombe* suggested that Dis3 is also required for correct chromosome segregation.

**Methodology/Principal Findings:**

We show here that the progression of mitosis is arrested in *dis3-54*, and that segregation of the chromosomes is blocked by activation of the mitotic checkpoint control. This block is dependent on the Mad2 checkpoint protein. Double mutant and inhibitor analyses revealed that Dis3 is required for correct kinetochore formation and function, and that this activity is monitored by the Mad2 checkpoint. Dis3 is a member of the highly conserved RNase II family and is known to be an essential subunit of the exosome complex. The *dis3-54* mutation was found to alter the RNaseII domain of Dis3, which caused a reduction in ribonuclease activity *in vitro*. This was associated with loss of silencing of an *ura4^+^* reporter gene inserted into the outer repeats (*otr*) and central core (*cnt* and *imr*) regions of the centromere. On the other hand, centromeric siRNA maturation and formation of the RITS RNAi effector complex was normal in the *dis3-54* mutant. Micrococcal nuclease assay also suggested the overall chromatin structure of the centromere was not affected in *dis3-54* mutant.

**Conclusions/Significance:**

RNase activity of Dis3, a core subunit of exosome, was found to be required for proper kinetochore formation and establishment of kinetochore-microtubule interactions. Moreover, Dis3 was suggested to contribute to kinetochore formation through an involvement in heterochromatic silencing at both outer centromeric repeats and within the central core region. This activity is likely monitored by the mitotic checkpoint, and distinct from that of RNAi-mediated heterochromatin formation directly targeting outer centromeric repeats.

## Introduction

Accurate chromosome segregation during mitosis is an essential event for proper cell multiplication. A large number of the genes required for different aspects of chromosome segregation have been identified. This includes genes encoding proteins implicated in centromere/kinetochore structure and function, sister chromatid cohesion, and chromosome condensation, together with proteins required for the spindle assembly checkpoint, activation of APC (anaphase promoting complex)/C (cyclosome) for ubiquitin-dependent proteolysis, and for anaphase promoting proteolysis that leads to the destruction of securin and the activation of separase [1]. In addition, various protein kinases such as Cdc2, polo and aurora and related protein phosphatases are implicated in mitotic control. These essential genes for chromosome segregation are conserved throughout eukaryotic organisms.

A series of fission yeast *Schizosaccharomyces pombe* cold sensitive mutants have been previously isolated that showed severe defects in sister chromatid separation at 20°C [2]. These mutants, termed *dis1, dis2*, and *dis3 (defective in sister chromatid disjoining)*, also contain hyper-condensed chromosomes at the restrictive temperature. Dis1 is a microtubule binding protein that is required for the proper attachment of kinetochore microtubules to the kinetochores, and is localized at the kinetochore specifically during mitosis [3], [4]. Dis1 is also the founding member of the Dis1-XMAP215-chTOG family of microtubule-associating proteins that are universally present in eukaryotes. Dis2 is one of the two catalytic subunits (Dis2, Sds21) of Type 1 protein phosphatase PP1. The *dis2-11* mutation is semi-dominant and blocks sister chromatid separation at the restrictive temperature [5]. Both Dis1 and Dis2 proteins are phosphorylated and their activities are regulated by Cdc2 kinase [6], [7]. Cdc2 down regulates the phosphatase activity of Dis2 and up regulates the kinetochore localization of Dis1 by direct phosphorylation during mitosis.

Unlike Dis1 and Dis2, the role of Dis3 in proper kinetochore function or mitotic progression is not clear. Dis3 is a relatively large protein (110 kD) that includes the characteristic RNase II (RNB) domain, which is highly conserved in bacteria, archaea and eukaryotes. RNase II contains a highly processive 3′→5′ exoribonuclease activity, and eukaryotic Dis3 homologs (Rrp44 in budding yeast) were identified as a component of the exosome that is required for RNA maturation and turnover in both the nucleus and cytoplasm [8]–[10]. Rrp44/Dis3 is one of more than ten protein components in the exosome, which can act upon different RNA substrates including mRNA, snRNA, snoRNA, rRNA and tRNA. The structure of *E. coli* RNase II was recently determined [11], [12] and revealed that the active site in the RNB domain is composed of four Asp residues and one Arg residue in two respective motifs referred to as Motif I and Motif IV. Mg^2+^ ion is chelated by the first and fourth asp residues in Motif I.

The human homologue of Dis3 was identified as an oncogene [13], consistent with a potential role in chromosome segregation and/or mitotic progression. Human Dis3 is tightly bound to Ran a small G protein [14]. Fission yeast Ned1, a homologue of human disease gene product lipin, directly interacts with Dis3, Pim1/Rcc1 (GEF for Ran) and nuclear pore proteins, and is implicated in complex cellular functions including chromosome organization and nucleo-cytoplasmic transport [15]. The Dis3 protein is located in both the nucleus and cytoplasm throughout the cell division cycle, and deletion of the *dis3^+^* gene in fission yeast showed that it is essential for cell viability [16]. The mitotically arrested phenotype is thus specific to the *dis3-54* mutant allele upon shifting to the restrictive temperature.

In order to determine how Dis3 contributes to mitotic progression, and more importantly, whether this represents a role for Dis3 ribonuclease in kinetochore formation/function, we have focused on the *dis3-54* mutant of fission yeast. Here, we report that the *dis3-54* mutation resides in the Dis3 RNB domain, and that the mutant protein contains a reduced RNase activity. We show that the spindle-kinetochore interaction is abnormal in *dis3-54* mutant cells and that this is associated with mitotic arrest at a pre-anaphase stage, which is dependent on the essential spindle assembly (mitotic) checkpoint protein, Mad2. We also show evidence that Dis3 is involved in a heterochromatic silencing process within the centromere.

## Results

### Arrested *dis3-54* mutant cells show an elongated metaphase-like spindle

**Figure 1:**
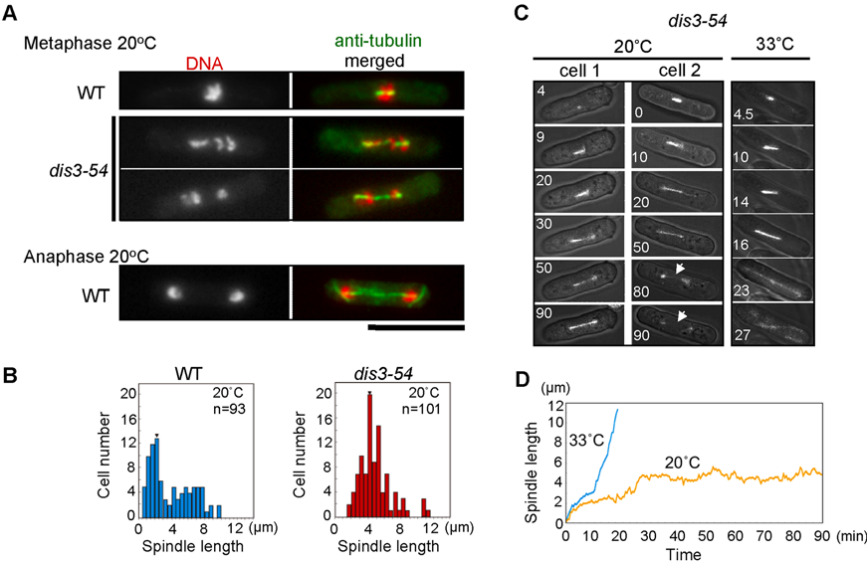
Elongated metaphase-like spindle in arrested *dis3-54* mutant cells. (A) Mitotic spindles in wild-type (WT) and *dis3-54* mutant cells at the restrictive temperature of 20°C. Cells grown in liquid culture were at 33°C were shifted to the restrictive temperature of 20°C for 4 hr and fixed with methanol. Anti-tubulin antibody TAT1 (green) and DAPI (red) were used for immunostaining and DNA staining, respectively. WT cells containing nuclei consistent with both metaphase and anaphase were observed as indicated, whereas *dis3-54* cells appeared to have arrested during mitosis with abnormally long metaphase-like spindles. Scale bar = 10 µm. (B) Mitotic spindle length distributions in WT and *dis3-54* mutant cells grown at restrictive temperature of 20°C for 4 hr. Cells were stained with anti-Sad1 (spindle pole body component) and anti-tubulin antibody after fixation with 3% paraformaldehyde and the pole-to-pole distance of the spindle microtubule in mitotic cells was measured. The inverted triangle represents the position of mode. (C) Time-lapse images of *dis3-54* mutant cells expressing chromosomally integrated GFP-tagged α2-tubulin (Atb2) under the inducible *nmt1* promoter. Images of cells growing at both 20°C (restrictive temperature, left panels) and 33°C (permissive temperature, right) were taken at 30 sec intervals for approx. 90 min with a confocal microscope. Numbers within each panel represent the time (min) after start of observation. The white arrow indicates the collapsed spindle microtubule, often observed after an extended period of mitotic arrest in *dis3-54*. (D) Time course plot of spindle length based on time-lapse images shown in Fig. 1*C*. Cells grown at 33°C (blue line) and 20°C (yellow line; “cell 1”) were measured and the 0 min time point was adjusted to reflect the start of mitosis. Normal changes in spindle length representing phases I, II and III were observed during the time-course at 33°C [18].

Cold-sensitive *dis3-54* mutant cells grown at 33°C were shifted to the restrictive temperature of 20°C and incubated for 4 hr to induce the nuclear division arrest phenotype. Arrested cells were fixed with methanol and the chromosomal DNA and microtubules were stained using DAPI and anti-tubulin antibodies, respectively. As expected, mutant cells displayed the *dis*-like phenotype [2], in that sister chromatids appeared not be separated and condensed chromosomes were scattered along the mitotic spindle (Fig. 1*A*). The average spindle length in *dis3-54* mutant cells arrested at 20°C (4.5 µm, Fig. 1*B*) was longer than that of the wild-type metaphase spindle (2.5 µm), but much shorter than the fully extended anaphase B spindle in wild-type (approximately 10–13 µm).

In order to monitor spindle changes over a period of time in actual living cells, a chromosomally integrated GFP-tagged α2-tubulin gene [17] was introduced into *dis3-54*. Mutant cells expressing GFP-tubulin both at restrictive (20°C) and permissive (33°C) temperatures were examined using a confocal microscope system equipped with a temperature control unit. Time-lapse images of cells cultured at 20°C and 33°C were obtained (representative cells shown in Fig. 1*C*; Movies S1, S2 and S3) and used for quantitative measurements of spindle lengths following initiation of mitosis (Fig. 1*D*). At the restrictive temperature of 20°C, phase 1 (period of spindle formation; [18]) occurred normally in *dis3-54* cells with an initial spindle length of 2.5–3.0 µm, however, the cells then appeared to remain in phase 2 (constant spindle length period; metaphase) as the spindle slowly reached a length of 4–6 µm within 90 min. Phase 3 (spindle extension period; anaphase B) did not take place and the spindle often collapsed after prolonged incubation at 20°C (indicated by white arrows in Fig. 1*C*). In contrast, *dis3-54* cells grown at the permissive temperature (33°C) displayed normal spindle elongation and completed anaphase with a long anaphase B spindle within 30 min after entry into phase 1.

**Figure 2:**
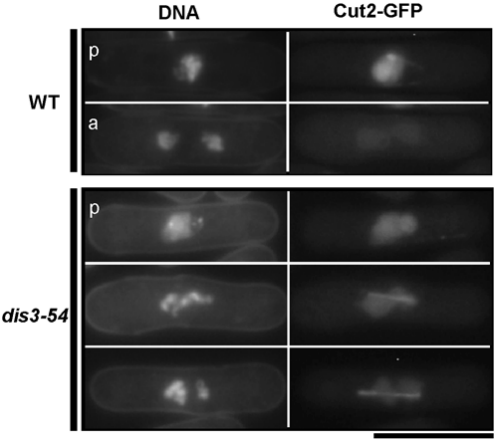
Mitotic arrest in *dis3-54* cells occurs at a pre-anaphase stage. WT and *dis3-54* mutant strains that carried a chromosomally integrated Cut2/securin-GFP fusion gene under control of the native promoter were cultured at 20°C for 4 hr in synthetic EMM2 medium. Cells were stained with Hoechst H33342 for visualizing DNA together with GFP signals, and were not subject to fixation. WT cells in both pre-anaphase (p) and anaphase (a) were observed, whereas persistent Cut2 signals in *dis3-54* (three representative cells shown) confirmed they were in a pre-anaphase stage. Scale bar = 10 µm.

To determine whether *dis3-54* cells cultured at 20°C were arrested at a pre-anaphase stage or following the switch to anaphase, the *dis3-54* mutation was combined with a chromosomally integrated copy of a GFP-tagged Cut2/securin gene under the control of its native promoter. Cut2/securin acts to prevent sister chromatid separation during metaphase and is degraded upon the triggering of anaphase [19]. In wild-type cells, Cut2-GFP signals were associated with condensed chromosomes during metaphase and disappeared in all anaphase and post-anaphase cells. In contrast, *dis3-54* cells cultured at 20°C constantly showed intense Cut2-GFP signals that remained with the condensed chromosomes and did not disappear (Fig. 2). This confirmed that anaphase (sister-chromatid separation) did not occur and *dis3-54* cells were indeed arrested at a pre-anaphase stage. Taken together, the above data indicates that a pre-anaphase spindle is produced in arrested *dis3-54* cells grown at the restrictive temperature, which remains present for 60–80 min.

### Mitotic arrest of *dis3-54* involves the checkpoint protein Mad2

Correct progression through mitosis is essential for cell survival and it is therefore carefully monitored by several proteins that prevent completion of the mitotic cycle should a potentially lethal problem be encountered [20], [21]. Mad2 is known to restrain the mitotic progression by preventing the activation of ubiquitin-dependent anaphase promoting proteolysis. Although *dis3-54* cells were found to be arrested at a pre-anaphase stage, it was unclear whether this was due to the *dis3-54* mutation directly preventing sister chromatid separation, or due to *dis3-54* resulting in activation of the mitotic checkpoint to halt mitotic progression at the pre-anaphase stage.

**Figure 3:**
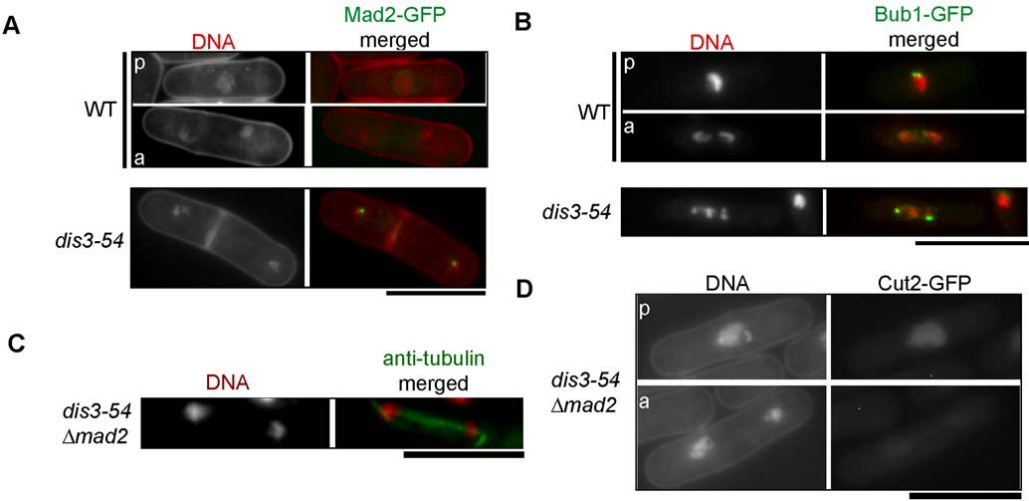
Pre-anaphase arrest in *dis3-54* is dependent on the spindle checkpoint protein Mad2. (A) Cellular localization of Mad2-GFP in WT and *dis3-54* mutant cells at 20°C. WT and *dis3-54* strains carrying a chromosomally integrated copy of Mad2-GFP were shifted to 20°C for 8 hr prior to imaging. Cells were stained with Hoechst H33342 for DNA and were not subject to fixation. WT cells in both pre-anaphase (p) and anaphase (a) were observed. All mitotically arrested *dis3-54* mutant cells contained intense Mad2-GFP signals (n = 23). (B) Cellular localization of Bub1-GFP in WT and *dis3-54* at 20°C. WT and *dis3-54* strains carrying a chromosomally integrated copy of Bub1-GFP were cultured as in A, however, cells were first fixed in methanol prior to staining with Hoechst H33342. Bub1-GFP dot signals were observed in WT cells only during the pre-anaphase stage, whereas intense signals were observed in all mitotically arrested *dis3-54* mutant cells (n = 24). Scale bar = 10 µm. (C) Full mitotic spindle elongation and asymmetrical nuclei in a *dis3-54 Δmad2* double mutant as observed by immunostaining. Cells were grown and treated as in Fig. 1*A*. (D) Cellular localization of Cut2-GFP in the *dis3-54 Δmad2* double mutant at 20°C. Loss of Cut2 signals demonstrates progression from pre-anaphase (p) to anaphase (a) in the absence of Mad2, similar to that observed in WT (compare with Fig. 2).

To distinguish between these two possibilities, we first investigated the localization of two spindle assemble checkpoint proteins, Mad2 and Bub1, in wild-type and mutant cells using chromosomally integrated GFP-tagged versions of these genes driven by their native promoter. Intense kinetochore dot signals corresponding to Mad2-GFP were observed in most *dis3-54* cells arrested at 20°C, whereas such signals were only normally seen in a small number of wild-type mitotic cells (Fig. 3*A*). Similar results were also obtained from the analysis of Bub1-GFP. In wild-type cells, Bub1-GFP was only observed in early mitotic stages, however. Intense kinetochore localization of Bub1-GFP was observed in most of the arrested *dis3-54* cells (Fig. 3*B*).

To confirm that the observed presence and kinetochore localization of Mad2 was functionally contributing to the mitotic arrest in *dis3-54*, the mutant cells were crossed with a Mad2 deletion mutant (*Δmad2*). In striking contrast to that of single *dis3-54* mutants, the spindle was fully extended in the double mutant cultured at 20°C (Fig. 3*C*) and similar to that observed in wild-type (see Fig. 1*A*). Analysis of GFP-tagged Cut2/securin localization in the double mutant also revealed loss of Cut2 associated with the appearance of daughter nuclei following pre-anaphase (Fig. 3*D*). This was similar to that observed in wild-type (see Fig. 2) and demonstrated that in the absence of Mad2, *dis3-54* mutant cells are able to overcome the arrest at pre-anaphase and proceed to anaphase. Two daughter nuclei that were asymmetrical in size or lagging chromosomes were often observed in the *dis3-54 Δmad2* double mutant cells, suggesting that although the spindle was extending normally, chromosome segregation was unequal. In both fission yeast and animal cells, the spindle checkpoint proteins Mad2 and Bub1 have been shown to be recruited to kinetochores that are not attached by kinetochore microtubules or in which the stable bipolar attachment is not established [22]–[24]. The above data strongly indicates that normal bipolar attachment between the kinetochore and spindle is impaired in *dis3-54* mutant cells, and the resulting spindle checkpoint inhibits the metaphase/anaphase transition.

### Sister chromatid separation and abnormal segregation in *dis3-54 Δmad2*

**Figure 4:**
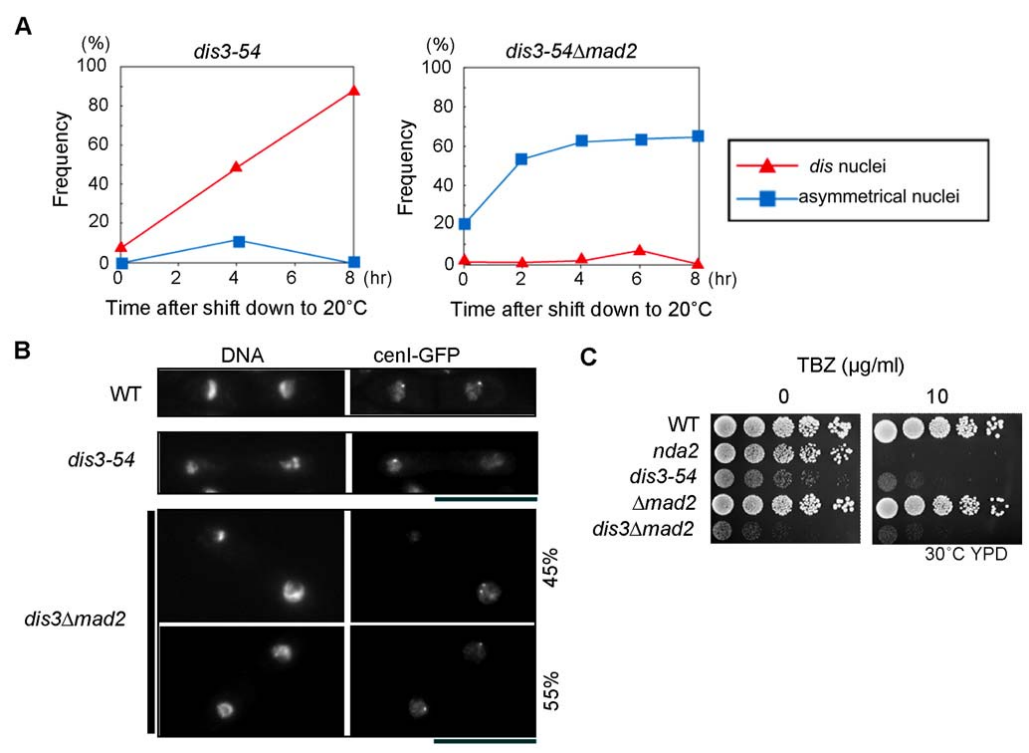
Removal of the mitotic checkpoint reveals unequal chromosome segregation in a *dis3-54 Δmad2* double mutant. (A) Time-dependent changes in frequency of different nuclei phenotypes observed in *dis3-54* single (left panel) and *dis3-54 Δmad2* double (right panel) mutants upon shift to the restrictive temperature of 20°C. Red line, *dis* nuclei phenotype (see Fig. 1*A*); Blue line, asymmetrical nuclei and lagging chromosomes (see Fig. 3*C*). (B) Localization of the CenI-GFP in the wild-type (WT), *dis3-54* single mutant and *dis3-54 Δmad2* double mutant cells. Cells were cultured and treated with Hoechst as in Fig. 1*A*. Sister chromatids were able to separate in the double mutant, but chromosome segregation was abnormal. (C) *dis3-54* and *dis3-54 Δmad2* mutants are hypersensitive to TBZ. Serial dilutions (1∶5) of each strain were spotted onto YPD solid media containing 0 and 10 µg/ml TBZ and incubated at 30°C. The cold- and TBZ-sensitive *nda2* mutant strain defective in α1-tubulin was used as control [25].

To investigate further whether sister chromatid separation could occur when the spindle checkpoint was compromised in *dis3-54* cells, the nuclear phenotype of both single *dis3-54* and double *dis3-54 Δmad2* mutants was examined over an extended time period following shift to the restrictive 20°C temperature. As shown in Fig. 4*A*, almost all single mutant cells contained the typical *dis*-like pre-anaphase nuclei (see Fig. 1*A*). In contrast, the proportion of cells with the “asymmetrical daughter nuclei” phenotype (see Fig. 3*C*) greatly increased in the double mutant cells and approximately 60% of the double mutant cells showed the asymmetrical nuclei phenotype by 4 hr after the shift to 20°C.

To directly observe how the sister centromeres were segregating in individual *dis3-54* and double *dis3-54 Δmad2* mutant cells, we crossed these mutants with a strain that expresses LacI-GFP and contains lacO repeats integrated at the *lys1* locus that is close to the centromere of chromosome 1 (*cen1*-GFP; [18]). All wild-type cells contained a single *cen1*-GFP spot at opposite ends of the cell corresponding to each copy of chromosome 1 (Fig. 4*B*), confirming normal segregation during anaphase. On the other hand, one or two *cen1*-GFP spots on the single pair of condensed chromosomes could be observed in the single *dis3-54* mutant cells arrested at 20°C, consistent with the notion that they were arrested at a metaphase-like stage. In the double mutant *dis3-54 Δmad2* cells, however, approximately 55% of anaphase cells showed the two segregated spots as seen in wild-type. The remaining double mutant cells contained two closely situated spots within the same daughter nucleus, suggesting that the sister chromatids had separated but did not move to opposite nuclei. This data indicates that although sister chromatid separation occurred in the double mutant upon loss of the mitotic checkpoint, chromosome segregation towards the opposite poles became abnormal, possibly due to lack of bi-orientation in paired sister centromeres that then moved to the poles in an apparently random fashion and caused the high frequency of unequal segregation.

### Hypersensitivity to a microtubule poison

Fission yeast mutants defective in kinetochore-microtubule attachment are usually hypersensitive to microtubule destabilizing drugs such as thiabendazole, TBZ [22], [25]–[28]. We examined whether this was also the case for the single *dis3-54* and double *dis3-54 Δmad2* mutants. Serial dilution of mutant cells were spotted onto YPD plates containing 0 and 10 µg/ml concentrations of TBZ, together with the control hypersensitive α1-tubulin mutant, *nda2* [29]. Both *dis3-54* and *dis3-54 Δmad2* were hypersensitive to TBZ even at the semi-permissive temperature of 30°C (Fig. 4*C*). The double mutant *dis3-54 Δmad2* also showed a recognizable synthetic effect, as the cells grew slowly in comparison with single *dis3-54* or *Δmad2* mutant cells.

### *S. pombe* Dis3 contains an RNase activity that is reduced by the *dis3-54* mutation

**Figure 5:**
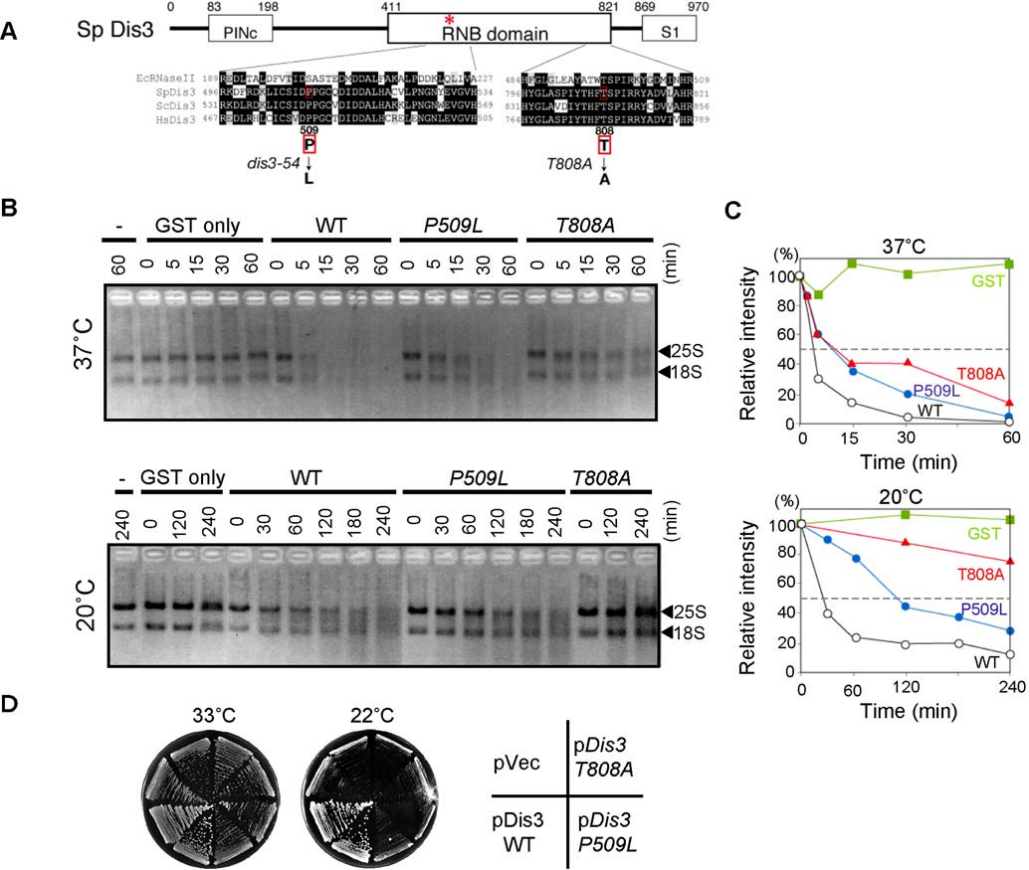
The *dis3-54* mutation alters the Dis3 RNase II domain and causes reduced RNase activity. (A) The Dis3 protein contains the PINc, RNB and S1 domains. PINc belongs to a large family of protein domains associated with exonuclease or nucleotide binding activity. S1 is an RNA binding domain [65]. The RNaseII RNB domain contains four high consensus regions, Motif I, II, III and IV [30]. Amino acid sequences from Motifs I and IV of *E. coli* (EcRNaseII), *S. pombe* (SpDis3), *Saccharomyces cerevisiae* (ScDis3) and *Human* (Hs) are compared. Identity and similarity are indicated as black and gray boxes, respectively. The *dis3-54* mutation alter the Pro509 residue to Leu (P509L) in Motif I, while another mutation affecting RNase activity (T808A) is located in Motif IV. (B) *In vitro* RNase activity of WT, P509L and T808A mutant Dis3-GST proteins at 37°C (top panel) and 20°C (bottom panel). Wild-type and mutant Dis3-GST proteins (1 pmol/lane) purified from *S. pombe* cells were incubated with total *S. pombe* RNA (1 µg/lane) as described in Materials and Methods. Reactions were stopped at the indicated time points and subject to denatured agarose gel electrophoresis. RNA was detected by ethidium bromide staining. 25S and 18S rRNA bands are indicated. (C) Quantitation of three independent experiments represented in Fig. 5*B*. Total ethidium bromide signal intensities for the major 25S and 18S rRNA bands were measured using an Image Analyzer (Bio-Rad) and normalized against the intensities for the 0 min time points. (D) A multicopy plasmid carrying the wild-type *dis3* gene is able to complement the cold sensitivity of *dis3-54* mutant at 20°C on YPD media, whereas equivalent plasmids carrying either the *dis3-54* (*P509L*) or *dis3-T808A* RNase mutant genes are unable to complement the cold sensitivity.

In order to further understand the role for Dis3 in mitosis, the causative mutation in *dis3-54* was first determined by sequencing the *dis3* gene in *dis3-54* mutant cells. A single nucleotide substitution was identified (C1690→T1690) that resulted in an amino acid substitution from Pro509 to Leu509 (Fig. 5*A*). Pro509 is located within the central region of Dis3 that contains a highly conserved RNB domain [30]. The RNB domain has been shown to be a catalytic module in RNaseII family proteins [11], [31] and Pro509 is conserved in all eukaryotic homologues, although the prokaryotic *E. coli* RNase II contains a serine residue at the equivalent position.

We next attempted to confirm that Dis3 does indeed contain ribonuclease activity, and if so, whether this is affected by the *dis3-54* mutation. Plasmids were constructed that contained either wild-type Dis3 (WT) or *dis3-54* mutant Dis3 *(P509L)* fused to a GST tag under control of the *nmt1* inducible promoter. Another residue in the RNB domain, Thr808, has been shown to be located in the catalytic region of RNB by crystallographic structure analyses [11], [12] and differs from Pro509 in that it is also highly conserved in prokaryotes, although its contribution to RNase activity was yet to be determined. We therefore targeted this residue as a control and constructed a third plasmid with RNB mutant Dis3 (T808A) fused to GST. After overproduction in fission yeast cells, GST fusion proteins were purified and *in vitro* RNase assays were performed at 37°C and 20°C using total RNA isolated from fission yeast as the substrate [8].

When the substrate RNA was incubated with control GST protein only, the RNA remained stable throughout the reaction periods. In contrast, the substrate RNA was rapidly degraded in a time-course dependent manner when incubated with wild-type Dis3-GST protein (Fig. 5*B*). This demonstrates that fission yeast Dis3 is indeed a ribonuclease that exhibits activity towards at least rRNAs *in vitro*. The RNase activity of wild-type Dis3 was somewhat slower at 20°C compared to that at 37°C, as most RNA was degraded within 15 min at 37°C compared to substrate RNA still remaining by 60 min at 20°C.

The *dis3-54* (P509L) and RNB (T808A) mutant Dis3 proteins were assayed in a similar manner to determine whether these mutations alter Dis3 RNase activity. Time-course analysis of substrate RNA degradation at 37°C showed that the P509L substitution result in reduced RNase activity of Dis3. In reactions containing wild-type Dis3, 50% of substrate RNA was degraded within 4 min at 37°C, whereas approximately 10 min was required for the same level of degradation with P509L and T808A mutant Dis3 (Fig. 5*C*). At 20°C, the effect of P509L and T808A mutations on RNase activity was more severe. Approximately 110 min was required for 50% degradation of substrate RNA by P509L mutant Dis3, in contrast to only 26 min for wild-type Dis3. The T808A mutation severely disrupted Dis3 activity as more than 50% of the target RNA still remained after 240 minutes.

The *dis3-54* mutation can be complemented with a plasmid containing a wild-type copy of the *dis3^+^* gene. To confirm that the reduction in RNase activity of Dis3 is directly linked to the *dis3* phenotype, we constructed equivalent Dis3 expression plasmid containing either the P509L or T808A mutations. As expected, P509L mutant Dis3 was unable to complement the *dis3-54* cold-sensitive (*cs)* phenotype. Expression of RNB mutant (T808A) Dis3 also failed to complement the *cs* phenotype, confirming the importance of Dis3 RNase activity for its kinetochore function (Fig. 5*D*).

### The *dis3-54* mutant contains a centromeric silencing defect

**Figure 6:**
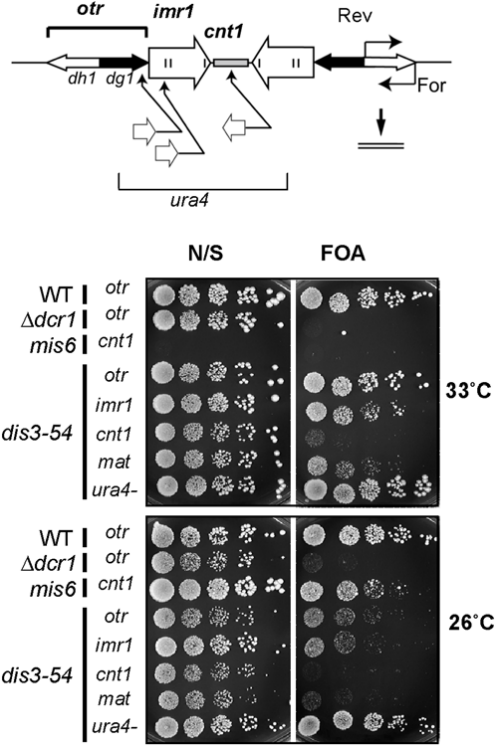
The *dis3-54* mutant shows defects in centromeric silencing. Centromeric silencing of four *S. pombe* strains, wild-type (WT), Dicer deletion RNAi mutant (*Δdcr1*), Mis6 kinetochore mutant (*mis6*) and single *dis3-54* mutant, was examined in a genetic background containing the *ura4* ^+^ reporter gene integrated into different regions of the centromere (*otr::ura4, imr1::ura4, cnt1::ura4*). Schematic diagram of *S. pombe* centromere I (upper panel) shows the *ura4^+^* insertion sites. A strain without the *otr::ura4* reporter was included as a control (*ura4* ^−^). Silencing of a reporter gene integrated into the mating type locus (*mat1)* was also examined for *dis3-54*. Serial dilutions (1∶5) of each strain were plated on YPD media without selection (N/S) and on YPD media containing 5-FOA (inhibitor of Ura+ strains), and incubated at permissive (33°C) and restrictive (26°C) temperatures as indicated. Note that *mis6* is a heat-sensitive mutant and that the permissive and restrictive temperatures for *mis6* are 26°C and 33°C, respectively.

Formation of *S. pombe* centromeric heterochromatin requires a pathway involving RNA metabolism [32]–[35]. To examine whether Dis3 may play a role in this pathway, centromeric silencing in *dis3-54* mutant cells was first tested. The fission yeast centromere consists of a central core region (*cnt*) flanked by inverted repeat domains (*imr*) and outer heterochromatic repeats (*otr*) [36]. Silencing in the central core region involved factors distinct from that at the outer centromeric repeats [37], and also involves kinetochore proteins such as Mis6, Cnp1, Mal2, and Sim4 [38], [39]. The *dis3-54* mutation was combined with *ura4^+^* marker genes inserted into either the heterochromatic *otr* region, inner *imr1* region, or central core *cnt1* regions (*otr, imr1, and cnt1::ura4* ^+^, see Fig. 6). Silencing of the *ura4* ^+^ reporter gene in the centromere context was examined. Control strains included a Dicer deletion RNAi mutant that is defective in heterochromatin silencing and formation (*Δdcr1 otr::ura4^+^*; [32]), a *mis6* kinetochore mutant that is defective in central core silencing at 33°C (*mis6 cnt1::ura4* ^+^; [39]), wild-type Dis3 containing the reporter construct (WT *otr::ura4^+^*), and a *dis3-54* mutant lacking the *ura4* gene completely (*dis3-54 ura4^−^*) .

Expression of the integrated *ura4^+^* gene was assayed by spotting serial dilutions of each strain on either non-selective media (N/S) or counter-selective media containing FOA (inhibitor of growth of Ura4^+^-expressing cells). The *otr::ura4^+^* construct was normally silenced in the wild-type strain such that cells could grow in the presence of FOA, whereas growth of the control non-silenced strain *Δdcr1 otr::ura4^+^* was inhibited by FOA (Fig. 6). In *dis3-54* mutant cells at 26°C (the semi-permissive temperature), silencing at the outer *otr* repeat and inner *imr1* regions was clearly reduced as FOA inhibited colony formation of *dis3-54 otr::ura4* ^+^ and *dis3-54 imr1::ura4* ^+^. The *ura4^+^* gene inserted at *cnt1* central core region was also desilenced in the *dis3-54* mutant at the semi-permissive 26°C. This silencing defect was more severe than that at the *imr1* and *otr* regions, and could be observed even at the permissive 33°C (Fig. 6). The Dis3 protein therefore has a role in silencing in all three major centromeric regions. In addition to the centromeric silencing defect, we found that silencing at the heterochromatic mating type locus (mat) was also reduced in the *dis3-54* mutant (Fig. 6).

### Native transcripts from outer centromeric repeats accumulate to low levels in the *dis3-54* mutant

**Figure 7:**
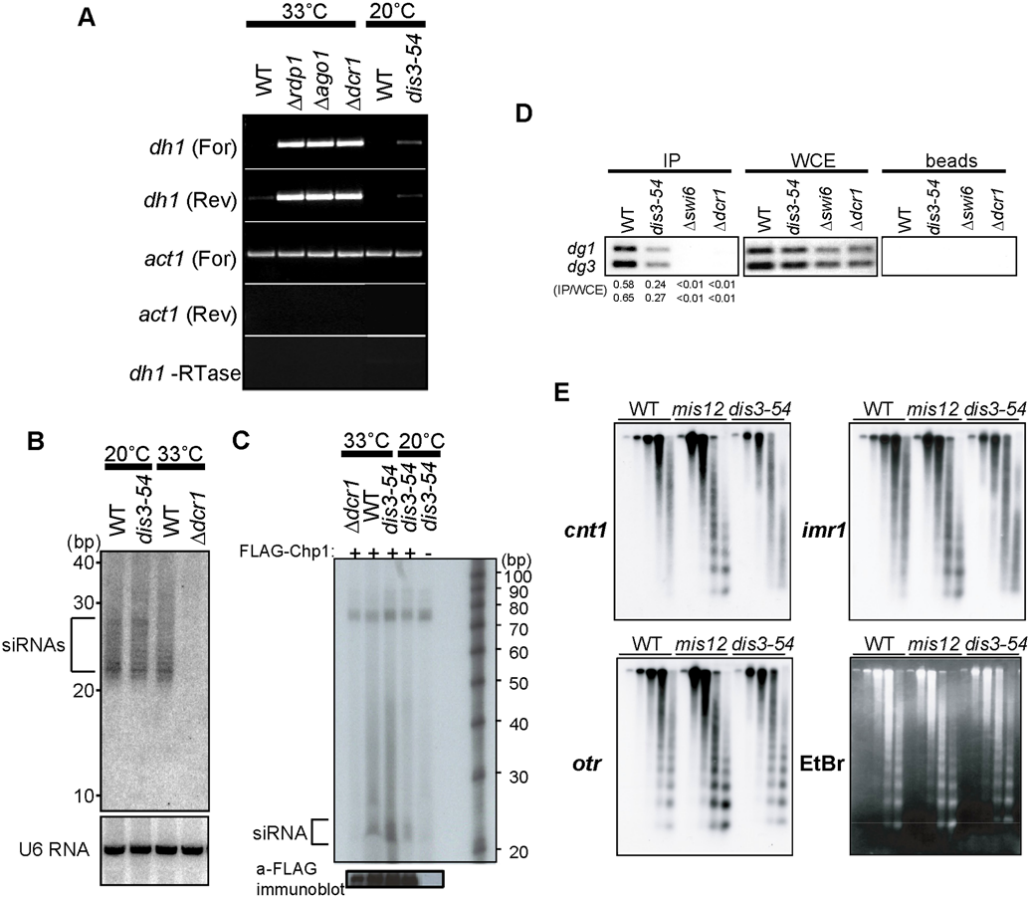
Centromeric siRNA production and and overall chromatin structure in *dis3-54* is similar to wild-type. (A) Accumulation of RNA transcripts derived from native centromeric repeats was determined by semi-quantitative RT-PCR using strand-specific primers. Total RNA isolated from WT and *S. pombe* RNAi deletion mutants, *Δrdp1, Δago1* and *Δdcr1*, grown at 33°C, and from WT and *dis3-54* cultured at the restrictive temperature (20°C) were assayed. Actin was also assayed as a loading control, and reactions without reverse transcriptase (-RTase) were included as a control against DNA contamination. (B) Total small RNAs were extracted from *S. pombe* wild-type (WT) and *dis3-54* mutant cells cultured at the restrictive temperature (20°C). WT and *Δdcr1* deletion mutant strains cultured at 33°C were included as a positive control. Small RNA northern blots were probed with a specific probe to detect siRNAs derived from the centromeric *dh* repeat. Position of RNA size marker is shown to the left. Blots were re-probed with U6 snRNA for loading control. (C) The RITS complex was isolated from wild-type (WT), *Δdcr1* and *dis3-54* mutant cells by immunoprecipitation of the FLAG-tagged Chp1 component. Small RNAs that co-purified with FLAG-Chp1 were extracted by phenol-chloroform and separated on a denaturing polyacrylamide gel after labeling with [5′-^32^P] pCp using RNA ligase. Position of the RNA size marker is shown to the right. (D) Localization of Swi6 protein at centromeric *otr* region in the *dis3-54* mutant. ChIP analysis was performed using antibody against Swi6 protein [58]. Co-precipitated DNA was purified and PCR amplified using *otr* (*dg1* and *dg3*) region specific primers. (E) Chromatin structure at the three centromere regions, *cnt1, imr1* and *otr* (*dg1*) in WT, *mis12* and *dis3-54* mutants. Nuclear chromatin fractions were prepared from WT and *dis3-54* mutant cells grown at 20°C for 8 hr, and *mis12* at 36°C (restrictive) for 8 hr, and digested with MNase for 0, 1, 2, 4 and 8 min. Southern Hybridization was then performed using specific probes for *cnt1, imr1* and *dg1*.

RT-PCR was employed to directly detect transcripts from the *otr* region in the *dis3-54* mutant. Specific primers were used to distinguish between both forward and reverse transcripts derived from the *dh* sequence of the *otr* repeats (see Fig. 6). In control RNAi mutant strains *Δdcr1, Δago1* and Δ*rdp1*, strong accumulation of transcripts from both the forward and reverse strands were observed, whereas only the reverse strand was just detectable in wild-type (Fig. 7*A* and [32]). In *dis3-54* cells grown at the restrictive temperature, accumulation of both the forward and reverse strands was observed, although this was weak and much less than that for RNAi mutants. Loss of centromeric silencing in *dis3-54* was nonetheless significant as no transcripts could be detected in wild-type cells grown under the same conditions.

### Centromeric siRNA production and incorporation into RITS is not affected in the *dis3-54* mutant

RNAi-mediated centromeric heterochromatin formation in *S. pombe* involves the formation of 22 ∼ 26 nucleotide (nt) small interfering RNAs (siRNAs) by Dcr1-dependent cleavage of centromeric dsRNAs derived from the *otr* repeats [32], [40]. These siRNAs are also incorporated into the effector complex called RITS, which is required for the downstream histone modification within the *otr* region [33]. In order to examine the role, if any, of Dis3 protein in siRNA production or incorporation into RITS, accumulation of centromeric siRNAs was first determined in *dis3-54* mutant cells.

Small RNAs were purified from *S. pombe* wild-type, *Δdcr1* deletion and *dis3-54* mutant cells grown at 33 or 20°C, and subject to Northern blot analysis using a probe specific to the *dh* region. As shown in Fig. 7*B*, siRNA production in the *dis3-54* mutant grown at the restrictive temperature was similar to that in wild-type and was not impaired. As expected, no siRNA was detected in the negative control *Δdcr1* mutant strain.

We next examined whether the siRNAs were normally incorporated into the RITS complex in *dis3-54* mutant cells. The *dis3-54* mutant was crossed with an *S. pombe* strain that carried the chromosomally integrated Flag-tagged Chp1 protein, which is a chromo-domain protein and a component of the RITS complex [33], [41]. Chp1-Flag was purified from cells grown at either 33°C or 20°C by immunoprecipitation, and analyzed for co-precipitated siRNAs after labeling with [5′-^32^P] pCp and RNA ligase [33]. In *dis3-54* mutant cells, small RNAs coprecipitated with Chp1-FLAG at both 20°C and 33°C similar to that in wild-type cells, although the association may be slightly weaker at the restrictive temperature (Fig. 7C). Consistent with previous published data, no small RNAs were associated with RITS in control *Δdcr1* cells [33]. The above data demonstrates that centromeric siRNAs are produced normally and are bound to RITS in *dis3-54* cells grown at the restrictive temperature.

### Swi6 protein remains associated with the *otr* region at reduced levels in *dis3-54*

Heterochromatin structure at the *otr* region in *S. pombe* centromeres is characterized by specific histone modifications and the subsequent binding of Swi6, which is a homolog of the mammalian heterochromatin protein 1 (HP1) and involved in heterochromatin spreading [42]–[45]. The presence of Swi6 at the outer repeats in the *dis3-54* mutant at the restrictive temperature was therefore examined using the chromatin immunoprecipitation (ChIP) assay. As previously reported, strong binding of Swi6 to the *otr* region was detected in WT cells, whereas no binding was observed in control *Δdcr1* RNAi mutant cells and a *Δswi6* mutant. In contrast, Swi6 remained associated with the *otr* region in the *dis3-54* mutant, although at a reduced level compared to that in wild-type (Fig. 7D). This result is consistent with the slight accumulation of transcripts from *dh* repeats in *dis3-54*. The fact that Swi6 was still localized at the outer repeats also supports the above finding that the mechanism for RNAi-mediated heterochromatin formation is not affected in the *dis3-54* mutant.

### Overall centromeric chromatin structure in the *dis3-54* mutant is similar to that in wild-type

The central core region of the centromere is known to have a specific unique chromatin structure, which is observed as a smeared pattern following micrococcal nuclease (MNase) digestion as opposed to a regular nucleosomal ladder pattern observed for the outer repeat region [36], [46]. Several mutants defective in silencing at core centromere, such as *mis6, mis12, cnp1, mal2* and *sim4*, have also been shown to be defective in maintenance of the specific central core chromatin structure [38], [39], [47]–[49]. We therefore used the MNase assay to investigate overall chromatin structure at the three major centromeric regions in the *dis3-54* mutant. As previously reported, the smeared pattern for *cnt1* and *imr1* regions in wild-type was lost in the *mis12* kinetochore mutant and changed to a regular pattern (Fig. 7E), consistent with the disruption of chromatin structure at core regions [48]. On the other hand, smeared MNase digestion for *cnt1* and *imr1* regions was maintained in the *dis3-54* mutant. At the outer repeats, the regular pattern observed in wild-type was also maintained in *dis3-54*. This data suggests that although Dis3 has a role in centromeric silencing, it is not required for overall centromeric chromatin structure.

### Synthetic lethality of *dis3-54* mutation with *Δswi6* or *Δdcr1*

**Figure 8:**
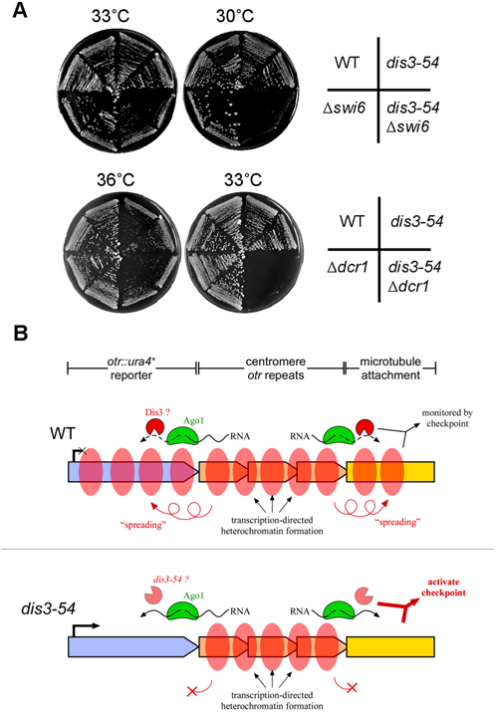
Dis3 may act downstream of RNAi in heterochromatic silencing and correct kinetochore formation. (A) Synthetic lethality of *dis3-54* mutant with the deletion strains of Swi6 (HP1 homolog; *Δswi6*) and Dicer (RNAi component; *Δdcr1*). Single and double mutants were plated on solid media and replica plates incubated at a range of temperatures from permissive to restrictive (36°C, 33°C, 30°C, 20°C). Synthetic defects were observed at semi-permissive temperatures for the double mutants as indicated. (B) One model for the role of Dis3 in correct kinetochore formation and function. Dis3 exosome activity is involved in heterochromatin silencing at regions neighbouring centromeric repeats, possibly processing transcripts cleaved by Ago1. This processing mechanism would also be important for establishment of a functional kinetochore and microtubule attachment. Dis3 activity (either directly or indirectly) is then monitored as a key step by the mitotic checkpoint, preventing serious errors in the absence of correct kinetochore formation.

Due to the fact that silencing of centromeric heterochromatin was defective in the *dis3-54* mutant, particularly in the *otr:: ura4^+^* region that is silenced by a spreading mechanism from neighbouring centromeric repeats, we examined whether the *dis3-54* mutation genetically interacts with heterochromatin mutations such as Δ*swi6* and Δ*dcr1*. Swi6 is a fission yeast homolog of mammalian heterochromatin protein HP1 that is involved in heterochromatin spreading [42]–[45] downstream of RNAi-mediated heterochromatin formation involving Dcr1. As shown in Fig. 8*A*, both the double mutants *dis3-54 Δswi6* and *dis3-54 Δdcr1* were synthetic lethal at 30 and 33°C, respectively, whereas each of the individual single mutants could produce colonies at the same temperature. The result suggested that the Dis3 protein might share an essential function with Swi6 and Dcr1 in heterochromatin formation or heterochromatin function during mitosis.

## Discussion

In this study, we show that the *S. pombe* cold-sensitive *dis3-54* mutation results in a single amino acid change (Pro509→Leu; P509L) within the characteristic RNase II RNB domain of Dis3 and causes a Mad2-dependent mitotic arrest at the restrictive temperature. Arrested cells show hyper-condensed mitotic chromosomes that are scattered along a metaphase-like spindle, which is longer in length than that normally observed during metaphase in wild-type. Continuous localization of the GFP-tagged spindle checkpoint proteins Mad2 and Bub1 at the kinetochores of arrested *dis3-54* cells suggested activation of the mitotic checkpoint. Consistent with this, deletion of the *mad2^+^* gene in *dis3-54* led to a release of the mitotic arrest and revealed unequal separation of sister chromatids and centromeres, strongly suggesting that the mitotic checkpoint was induced by a failure in proper kinetochore-spindle interaction. The segregation phenotype of the *dis3-54 Δmad2* double mutant resembles that of other centromere/kinetochore mutants in *S. pombe* [38], [47]–[49], and the elongated metaphase spindle in arrested *dis3-54* cells is a common kinetochore mutant phenotype in fission yeast [48], [50], [51]. Taken together with the hypersensitivity of *dis3-54* to the tubulin poison TBZ, the above data indicates that the kinetochore structure and function is impaired in *dis3-54* at the restrictive temperature.

In other eukaryotes, Dis3 has been identified as a component of the exosome that is involved in 3′→5′ RNA processing. However, to the best of our knowledge, no reports to date have identified a relationship between the RNase activity of Dis3 and proper mitotic progression. It is possible that Dis3 in fission yeast had evolved a kinetochore-specific function and that the RNase activity is either lost or distinct from this role. Our *in vitro* analyses confirmed that WT *S. pombe* Dis3 protein does indeed retain RNase activity. Moreover, the amino acid change caused by the *dis3-54* mutation, Pro509→Leu509, was found to cause reduced Dis3 RNase activity. The possibility that Pro509 is a specific residue required for a mitotic function independent of RNase activity was addressed by examining a second mutation in the RNB domain that is located almost 300 residues C-terminal of *dis3-54* and also reduced RNase activity (Thr808→Ala; T808A). Dis3 constructs containing mutations located outside the RNB domain are able to complement the cold-sensitive phenotype of *dis3-54* (H.M., M.Y., unpublished data), similar to the wild-type Dis3 gene. In contrast, Dis3 constructs containing the T808A RNB mutation and the wild-type Pro509 residue could not complement the cold-sensitive *dis3-54* phenotype. This strongly suggests that it is the correct RNase activity, rather than the actual Pro509 residue, that is required for the kinetochore function of Dis3.

Mitotic and chromosome segregation defects have been reported for three fission yeast mutants, *dhp1-1, pfs2-3169* and *Δcid14*, that are also defective in RNA metabolism. Dhp1 is a 5′→3′ exoribonuclease required for the processing and export of poly(A) RNA in the nucleus, whereas Pfs2 and Cid14 are involved in mRNA 3′-end processing and polyadenylation [52]–[54]. However, unlike for Dis3, these proteins are not directly related to the exosome. Nonetheless, the mitotic phenotypes of *dis3-54* and the above mutants do indicate significant roles for RNA processing in correct kinetochore function and chromosome segregation.

How might the exosome activity of Dis3 contribute to kinetochore formation, such that when this activity is compromised the mitotic checkpoint is activated? One possible explanation is that RNA metabolism of unknown target genes whose products are essential for kinetochore function is defective in *dis3-54* mutant. In this case, identification of accumulated RNAs in *dis3-54* mutant may be crucial for understanding the mechanism of mitotic arrest in *dis3-54* mutant. Another plausible explanation suggested by our data is through a possible link with heterochromatin silencing. In the *dis3-54* mutant, silencing was defective at reporter genes inserted into the outer repeats and central core of centromere. Both these regions of the centromere, especially the central core, are known to be essential for faithful segregation of chromosomes in mitosis [55].

At the central core region of the centromere, the *dis3-54* mutant showed a more severe silencing defect without a disruption of the overall specific chromatin structure itself. The conserved kinetochore proteins Mis6 and Mis12 were also normally localized at core regions in the *dis3-54* mutant (data not shown). This phenotype is similar to that in *alp5-1134*, a histone acetyltransferase component mutant, which was reported to be required for silencing at the central core [56]. The *alp5-1134* mutant also causes a chromosome segregation defect that results in activation of spindle checkpoint in mitosis [56]. Like the Alp5 protein, Dis3 may have an important function in silencing at core centromere to ensure the faithful kinetochore function, but not in the maintenance of the structure itself.

Centromeric transcripts derived from outer centromeric repeats are known to be processed by the RNAi machinery to mediate heterochromatin formation [32]–[34]. Recently, it was shown that heterochromatin spreading and silencing of reporter genes neighboring outer centromeric repeats requires an endonucleolytic cleavage (slicing) activity by the Ago1 RNAi component [57]. Significantly, the cleavage products resulting from wild-type Ago1 slicing activity accumulated in mutants that lacked the Rrp6 nuclear-specific exosome subunit, indicating that the nuclear exosome is involved in processing these transcripts. In the absence of Ago1 slicing activity, heterochromatin structure was only slightly reduced at the outer centromeric repeats, whereas silencing of the *otr::ura4* ^+^ reporter gene was lost. This is similar to the phenotype observed here for *dis3-54*. The Dis3 subunit was also recently shown to be responsible for core exosome activity and to have a nuclear function that partially overlaps with Rrp6 [58]. It is thus highly likely that Dis3 also processes centromeric transcripts, possibly even those cleaved by the Ago1 protein. Although further study is required, the above data support a hypothesis that the Dis3 activity is involved in a heterochromatin silencing process that may be linked to its role in the formation of a function kinetochore (Fig. 8B)

## Materials and Methods

### Yeast strains and culture media

Culture media used was YPD (1% yeast extract, 2%polypeptone, and 2% glucose). For plating, media was solidified with 1.5% agarose. *S. pombe* mutants *dis3-54, Δmad2, nda2, mis6, mis12, Δswi6, Δdcr1, Δrdp1* and *Δago1* were described previously [16], [22], [29], [32], [48], [59]. Parental *S. pombe* strains used for visualization of Cut2-GFP, Mad2-GFP, Bub1-GFP and cenI-GFP were also described previously [18], [22], [60].

### Immunofluorescence microscopy

Cell expressing GFP-tagged genes integrated on the chromosome were fixed with methanol and imaged using immunofluorescence microscopy as previously described [61]. The TAT1 antibody was used for tubulin immunostaining [62].

### In vitro RNase assays

The procedure for *in vitro* RNase assays was basically as according to [8]. Assays were performed using affinity-purified recombinant protein estimated by SDS-PAGE. Purity of the purified GST fusion proteins were confirmed by Coomassie blue staining (Figure S1). Assay were performed in 10 mM Tris-HCl at pH7.6, 50 mM KCl, 5 mM MgCl_2_, 10 mM DTT, 100 µg/ml BSA, 5.0 µg Yeast RNA and 0.8 U/µl RNasin; Promega. Reactions were stopped by addition of 1/10 vol of 8% SDS and 100 mM EDTA, and heated at 65°C for 15 min after adding 1 vol of 50% formamide and loading dye. Aliquots of 20 µl were resolved by agarose gel (1.2% agarose, 20 mM MOPS, 1 mM EDTA, 50 mM sodium acetate and 2.2 M formaldehyde) electrophoresis, and stained by ethidium bromide (EtBr). The quantity of EtBr stained RNA was assessed by Molecular Imager GS-700 (Bio-Rad).

### RNA analyses

Small RNA was prepared from cells growing exponentially in appropriate medium, and RT-PCR and Northern blots were performed as described [63]. Analysis of RITS complex containing siRNA was described previously [64].

### ChIP analysis

ChIP analysis was performed as described [47]. Cells cultured at 20°C for 8hr were fixed by formaldehyde. Sequence of primers used are:

For dg1: 5′-CTATAAATGGTTGACACAGC-3′ and 5′-CAAGCCAAGTCAGAGCAG-3′

For dg3: 5′-TGGTACCGAAGCACTGAC-3′ and 5′-ACTATCACTACTCTGAAGAC-3′

### MNase nuclease digestion

The MNase digestion assay was performed as described previously [47].

## Supporting Information

Figure S1 Supplemental figure.1
(8.74 MB TIF)

Movie S1 Tubulin-GFP living in dis3-54 at 20C #cell-1
(677 KB MOV)

Movie S2 Tubulin-GFP living in dis3-54 at 20C #cell-2
(858 KB MOV)

Movie S3 Tubulin-GFP living in dis3-54 at 33C
(296 KB MOV)
