# Supplementary figures and images for: Ribonuclease Activity of Dis3 Is Required for Mitotic Progression and Provides a Possible Link between Heterochromatin and Kinetochore Function
Source: PLoS One. 2007 Mar 21;2(3):e317. doi: 10.1371/journal.pone.0000317 (PMC1820850; doi:10.1371/journal.pone.0000317)

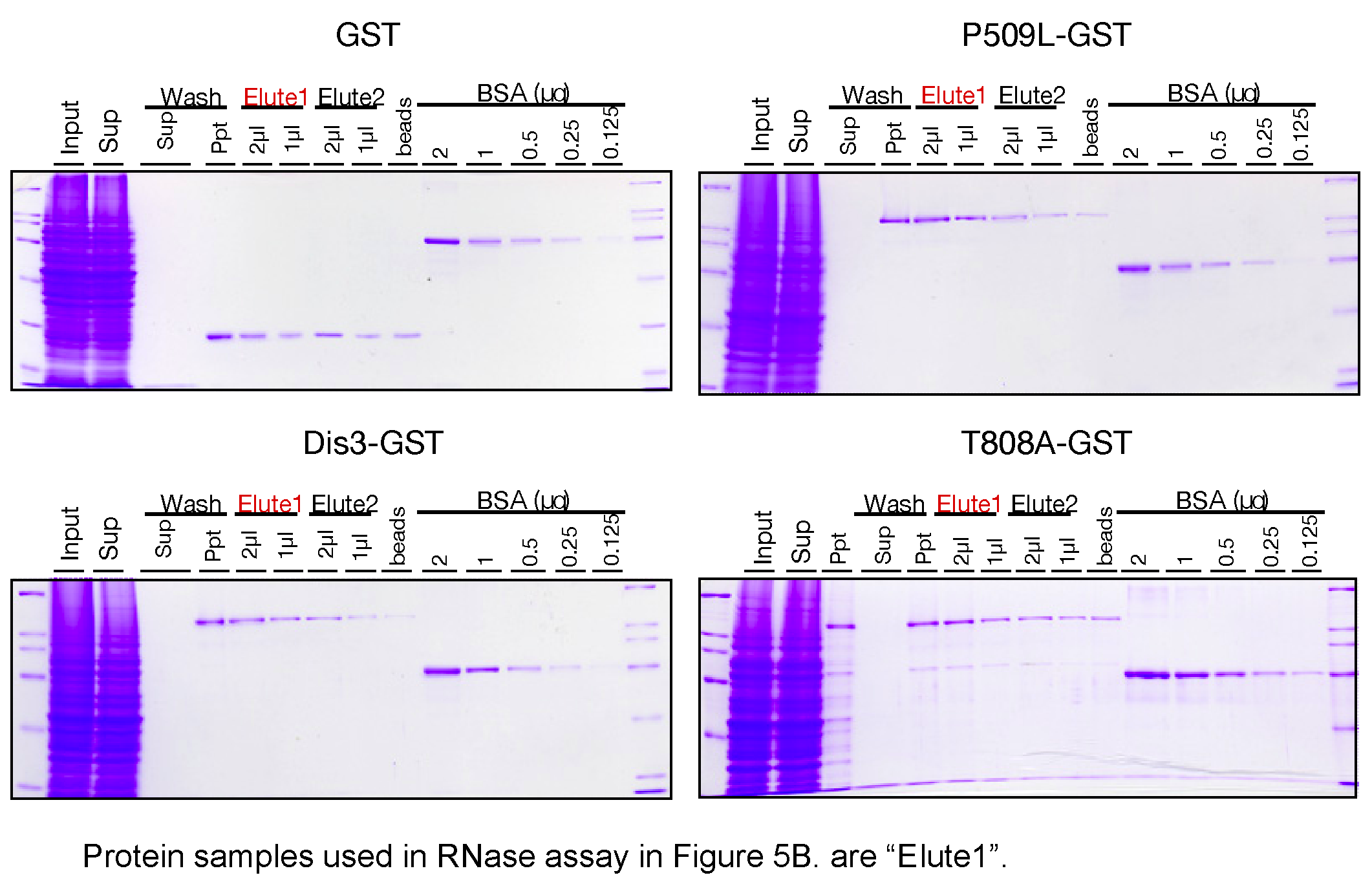

Supplement: Figure S1 — Supplemental figure.1 (8.74 MB TIF) [file pone.0000317.s001.tif]
